# Flow-driven patterns of whale shark movement in the Red Sea

**DOI:** 10.1038/s41598-026-45029-8

**Published:** 2026-04-02

**Authors:** Raquel L. Ostrovski, Jesse E. M. Cochran, Yuri Niella, Alkiviadis Kalampokis, Israel J. S. Filho, Ute Langner, Royale S. Hardenstine, Paula Moraga, Michael L. Berumen, Burton H. Jones

**Affiliations:** 1 Biological and Environmental Science and Engineering Division, King Abdullah University of Science and Technology, Thuwal, 23955-6900 Saudi Arabia; 2 IMOS Animal Tracking Facility, Sydney Institute of Marine Science, Mosman, NSW Australia; 3 Qualitas Instruments S.A, C. de la Toronga, 31, Madrid, 28043 Hortaleza Spain; 4 Computer, Electrical and Mathematical Sciences and Engineering Division, King Abdullah University of Science and Technology, Thuwal, 23955-6900 Saudi Arabia

**Keywords:** Eddies, Environmental variables, Habitat preferences, Megafauna movement, Habitat modeling, Saudi Arabia, Animal migration, Behavioural ecology, Ecological modelling

## Abstract

Dynamic ocean features (e.g., currents, eddies) can drive large-scale transport of water masses and nutrients, facilitating localized plankton blooms and affecting the migration patterns of higher trophic levels. The whale shark, *Rhincodon typus*, is a highly migratory planktivore known to aggregate in areas of ephemeral food abundance. While the species’ presence has been widely correlated with environmental variables such as water temperature and chlorophyll-a, the Red Sea population appears to be primarily associated with other oceanographic variables. Here, we investigated factors influencing juvenile whale shark presence within the Red Sea by correlating previously published tracking data with remote sensing measurements of environmental variables using Generalized Additive Mixed Models (GAMM). Model results revealed a significant correlation between whale shark presence and mixed-layer depth, wind direction, north-south current velocity, and temperature. The model trends and satellite-tracking analysis also indicated that whale sharks would spend more time actively following eddies within the basin. This new insight can help understand how whale sharks rely on ocean dynamics in this nutrient-poor subtropical basin, as regions influenced by wind, chlorophyll enrichment, and vertical mixing offer increased foraging opportunities for the species. It may also serve as a reference point for future research that identifies key whale shark habitats and considers the impact of climate change on preferred environments.

**Supplementary Information:**

The online version contains supplementary material available at 10.1038/s41598-026-45029-8.

## Introduction

Mapping the migration patterns of highly mobile marine organisms is often difficult due to the irregular and highly scattered nature of chance encounters^1^. The scarcity of movement ecology data can hinder conservation efforts, leaving migratory corridors and other critical habitats unprotected^2^. Unfortunately, tagging and tracking large marine animals can be both time-consuming and expensive^3–5^. One way to maximize the value of tracking data is to identify key environmental variables that may influence a species’ presence or absence, potentially guiding both research and management efforts without the need for additional tagging. For instance, the movement patterns of planktivorous megafauna are almost certainly driven, at least in part, by the patchy distribution of their prey^6,7^. Identifying the environmental factors influencing zooplankton distribution could be used to predict hotspots for large planktivores, especially in the tropics, where plankton blooms tend to be both spatially and temporally constrained^8^.

The whale shark (*Rhincodon typus*, Smith 1828) is a large, planktivorous elasmobranch that ranges widely throughout most tropical and subtropical seas^9,10^. Globally, whale shark aggregations tend to occur at coastal fronts, upwelling zones, and other oceanographic features associated with elevated phytoplankton biomass, reflected by increased chlorophyll-a concentrations, temperate sea surface temperatures (SST), and dynamic current systems^11–14^. These environmental parameters serve as reliable proxies for prey availability, guiding whale sharks to productive feeding grounds^10,15–17^. However, a unique aggregation site exists in the central Red Sea, around the Al Lith area^18,19^. This population displays seasonal migratory behavior that involves movement along the basin’s oligotrophic axis, occupying regions with generally low chlorophyll levels and distinct thermal and current regimes^18,20^. This apparent decoupling from the usual environmental cues that predict whale shark presence elsewhere highlights a knowledge gap. To date, the relationship between these environmental parameters and whale shark distribution in the Red Sea remains insufficiently evaluated, motivating the present investigation.

The Red Sea, a semi-enclosed, oligotrophic basin of the Indian Ocean, exhibits distinctive features, including high temperatures and salinity levels and no permanent freshwater input^21–24^. The movement of nutrients through this realm relies on a dynamic system of circulation that is influenced by geography and seasonality. The Gulf of Aden provides nutrient-rich waters that impact the central and southern parcels, while chlorophyll enrichment in the north relies on mechanisms such as wind patterns, deep convective mixing, and eddy-driven upwelling^25–27^. Monsoonal influences on temperature, salinity, and productivity in both northern and southern regions cause upwelling in summer and downwelling in winter^28^. Lastly, mesoscale features (i.e., fronts and eddies) present throughout the basin further influence the Red Sea’s productivity and ecosystem dynamics by creating conditions that concentrate nutrients and provide reliable food sources^29–31^.

Here, we employed an interdisciplinary approach to identify key environmental features that may influence the presence and movement of whale sharks within the basin. It is predicted that whale sharks likely have a non-random use of the Red Sea basin driven by differences in physical oceanographic conditions. Understanding the correlations between behavior and the environment is crucial for predicting potential changes in animal behavior and habitat preferences as the effects of climate change intensify. Whale sharks are currently classified by the IUCN Red List as “Endangered”^32^ and are under Appendix II of CITES. Therefore, a better understanding of the effects of oceanic features should be a key consideration when formulating legislation and policy for the worldwide conservation and management of this threatened species.

## Materials and methods

### Data collection

Whale shark tracking data were sourced from Berumen et al.^18^. This prior work deployed surface positioning and archival tags in 45 juvenile whale sharks (Wildlife Computers SPOT5 and MK10 Fastloc tags *n* = 19; MK10 tags *n* = 26), of approximately 5 m total length, from both sexes, during sporadic encounters between 2009 and 2011 in the central Red Sea region, near the coastal Saudi Arabian town of Al Lith (20.1405º N 40.2782º E). Daily environmental data were downloaded from the Bluelink Reanalysis Model (BRAN) database (CSIRO, Australia, available at: https://research.csiro.au/bluelink/outputs/data-access/). The BRAN model integrates multi-year data, incorporating remote sensing and in-situ observations to provide a realistic description of oceanic variables with global coverage. This database is particularly valuable when observational data is unavailable at daily resolution due to factors like cloud cover or transmission delays. Data on sea surface temperature (SST), sea surface height (SSH), mixed layer depth (MLD), wind speed (Wspd), wind direction (Wdir), vertical and horizontal current speed (VCS and HCS), north-south current velocity (VCUR), west-east current velocity (UCUR), and bottom-surface current velocity (WCUR) were obtained at 10 km spatial resolution. We followed previous research on physical parameters that directly influence whale shark presence^11–14^, but also took the liberty of adding extra variables that could potentially impact the Red Sea population based on local dynamics^25,27,29^. The environmental data were downloaded using the remora package in R Software (available at https://github.com/IMOS-AnimalTracking/remora).

### Data analysis

Move persistence (γ_t_) is a movement behavior index that ranges from 0 to 1 and indicates potential changes in animal movement patterns based on correlations between speed and direction^33^. A higher value of γ_t_ indicates more consistent movement (i.e., if an animal is undergoing a prolonged migration), while a lower value indicates less consistent movement (i.e., if an animal spends more time within a certain region). The R package aniMotum^33^ was used to model whale shark move persistence in the Red Sea. Shark locations from all 45 individuals were processed using a speed filter of 3 m/s and standardized for daily resolution (i.e., one location per day); a correlated random walk model was used to obtain the corresponding γ_t_ values for each position. A Generalized Additive Mixed Model (GAMM) analysis was used to investigate environmental factors affecting whale shark presence in the study region. For this analysis, we used solely data from both SPOT5 and MK10 Fastloc tags, considering the minimal error associated with their generated locations. We modeled ten environmental parameters (SST, SSH, MLD, Wspd, Wdir, VCS, HCS, VCUR, UCUR, WCUR) to assess their potential influence on the movements of tracked whale sharks. The aniMotum package was used to generate pseudo-absence locations for each shark, simulating plausible positions where empirical tracking data were unavailable. A 10-fold cross-validation was used for model validation using the “gamclass” package^34^. For each shark tracked, a total of 100 simulated tracks (Figure S1) were obtained using the original tagging location, step length, and distribution of turning angles as their corresponding observed tracks (i.e., the real tracks performed by the sharks during the monitoring). The simulated tracks were partitioned by randomly assigning the occurrence of each point throughout the track and the frequencies at which they occurred, while maintaining the actual number of individual steps for each shark. Additionally, the angles between points were randomized, ensuring no a priori expectations regarding the direction of shark movement. The model was programmed to incorporate a random turning angle if a step crossed a land border. Only the 25 simulated tracks most similar to the original tracks were kept for the analyses, as they were observed to be more correlated with the variations in the environmental data (Figure S2)^35,36^. Only consecutive locations separated by > 0.25º were selected and included in the model to reduce spatial and temporal autocorrelation and to minimize potential biases from pseudo-replication.

The GAMM was run with the “mgcv” R package^37^ using a binomial family of errors to learn the model parameters since the response variable comprised presence (i.e., the real tracks) and absence (i.e., the simulated tracks) data. Each candidate model included the shark identification number (ID) as a random effect to inspect for intra-individual movement variations. Multicollinearity was assessed with Pearson’s Correlation Test to identify high relations between environmental predictors. Predictors with Pearson correlation coefficients < 0.3 were retained together in the analysis (Figure S3). Variables were incrementally introduced in a stepwise fashion according to their higher AIC weights (Table 1), with retention contingent upon their ability to enhance model fit (i.e., a new model with higher AIC weight and significantly different from the previous simpler one, determined from an Analysis of Variance). The maximum degrees of freedom (k) for each variable was kept at 3 to avoid model overfitting. The final model was visually assessed to confirm a normal distribution of residuals through ANOVA plus the Chi-squared hypothesis test.

## Results

**Fig. 1:**
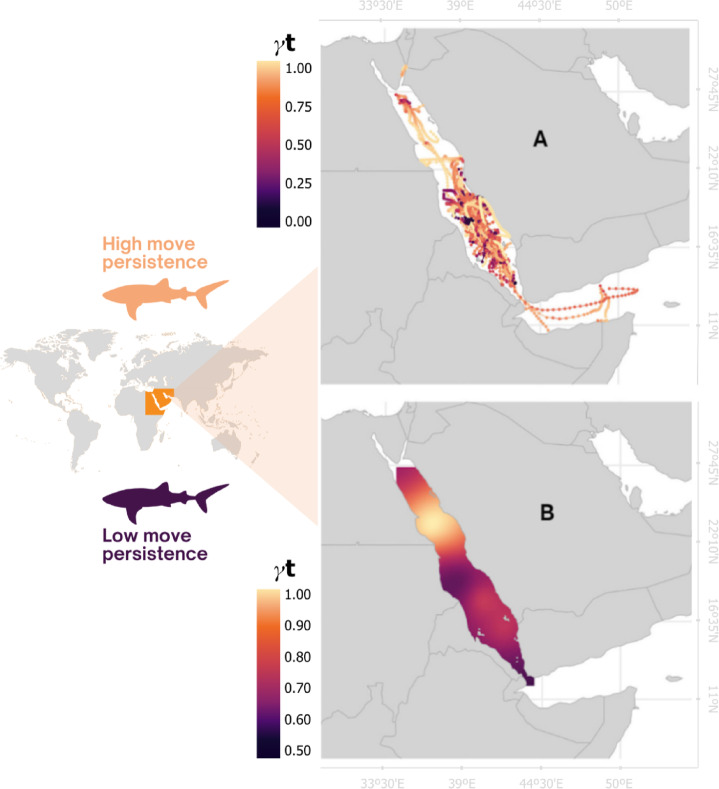
Move persistence (γ_t_) analysis for 45 whale shark tracks between 2009 and 2012 in the Red Sea basin. Higher values of γ_t_ indicate more persistency of movement and less time spent in an area. Lower values of γ_t_ reveal less persistence in movement, indicating more time spent within a region, which may reflect foraging or searching behavior. **A**: Move persistence analysis for all 45 tracks displayed individually; **B**: move persistence heatmap of all 45 tracks combined as a heatmap.

Whale sharks exhibited distinct movement patterns within the Red Sea (Fig. 1). A spatial heatmap indicated lower γ_t_ levels for the axis of the central area and within the southern region, showing lower movement persistence and indicating that the sharks may be spending more time actively searching or foraging. “Lower” in this context refers to values below the midpoint of this scale (0.5). The northern region yielded higher values of γ_t_, indicating a high movement persistence, i.e., a more transitional area. Few individuals (*n* = 2) moved persistently while traveling and then exhibited lower movement persistence in the northern Red Sea, suggesting a behavioral change from transiting to foraging or searching.

**Fig. 2:**
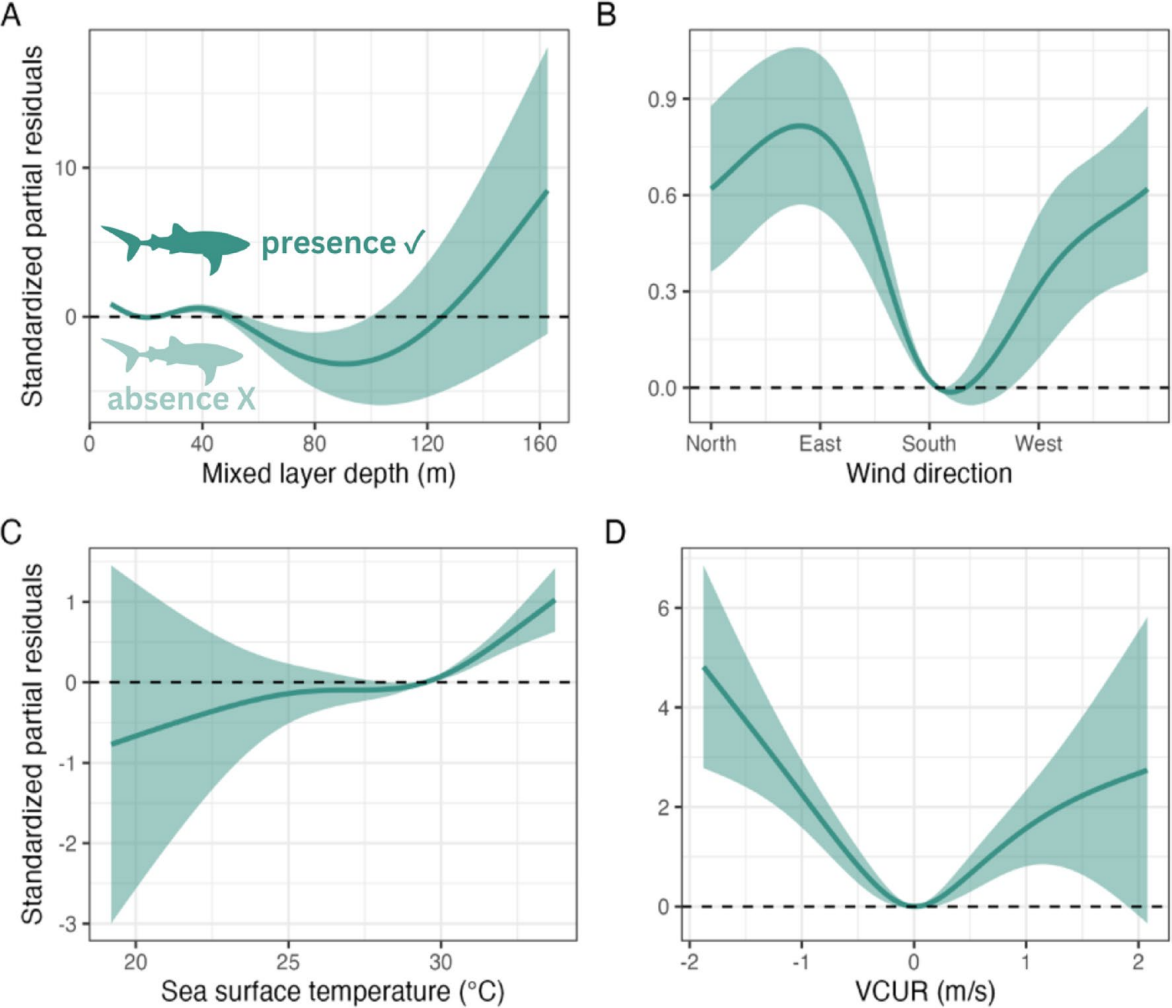
Generalized Additive Mixed Model (GAMM) of environmental variables found to significantly influence whale shark presence, including the effects of (**A**) mixed layer depth, (**B**) wind direction, (**C**) sea surface temperature, and (**D**) north-south current velocity on the x-axis. The y-axis represents the flexible functions of the variables in the model. The horizontal dashed lines and shaded areas represent the null effects and 95% confidence intervals, respectively. All values above the horizontal dashed line mean whale shark presence is significantly correlated with the respective variable tested, whereas all values below the line represent correlations with whale shark absence.

The effects of mixed layer depth, wind direction, sea surface temperature, and north-south current velocity were all significant in the environmental modelling (Table 1). GAMM results revealed that MLD showed the strongest effect (edf = 3.89, Ref.df = 3.99, χ^2^ = 94.73), followed by Wdir (edf = 2.71, Ref.df = 3.00, χ^2^ = 57.76), SST (edf = 2.69, Ref.df = 3.19, χ^2^ = 44.04), and VCUR (edf = 3.54, Ref.df = 3.89, χ^2^ = 53.07). The random effect of individual ID was significant (edf = 20.34, Ref.df = 25, χ^2^ = 210.84, *p* = 2e-16), indicating strong heterogeneity among animals. Partial residual plots revealed significant smooth effects of mixed layer depth, wind direction, sea surface temperature, and current velocity on the presence of whale sharks in the studied region (Fig. 2). Whale shark presence showed a bimodal trend with mixed layer depth, with positive values at 40 m and also areas deeper than 120 m (Fig. 2A). Wind direction also influenced presence, with higher probabilities observed under predominantly northwesterly winds, and minimal on southerly winds (Fig. 2B). Sea surface temperature was positively associated with whale shark presence, with occurrence increasing in warmer surface waters (Fig. 2C). Lastly, north-south current velocity showed a nonlinear effect, with less probability of shark presence in mild currents, but higher whale shark presence according to stronger southward (negative values) and northward (positive) currents speeds (Fig. 2D).

**Fig. 3:**
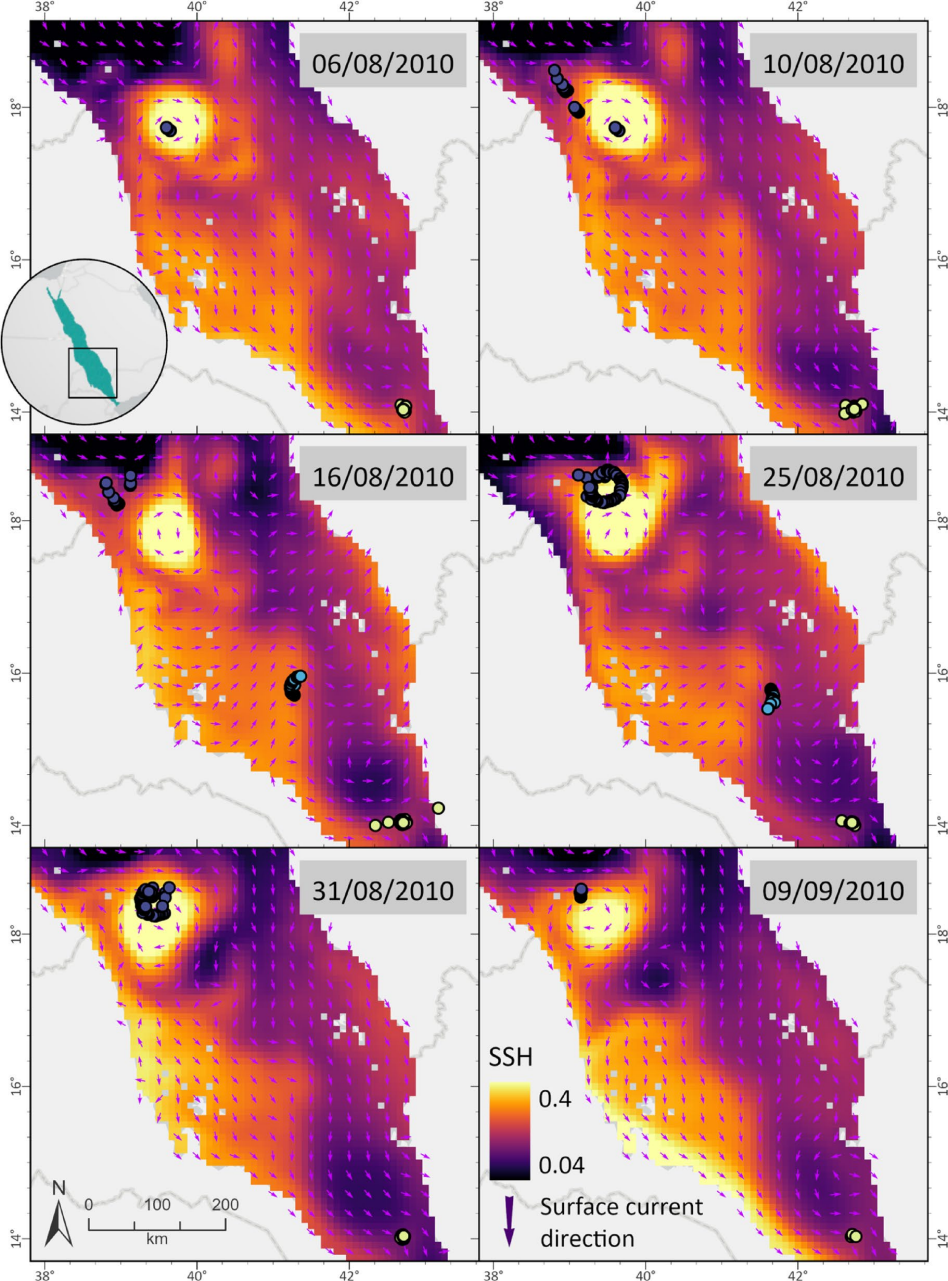
High-resolution (SPOT, MK10-FL) movement track data for individual whale sharks from August 6th until September 9th, 2010, grouped with sea surface height (SSH) circulation analysis for the Red Sea area. Individuals exhibited a preference for following both anticyclonic (bright yellow) and cyclonic eddy (dark purple) features along the basin, staying within its limits for several days. SSH data for the Red Sea was gathered from Copernicus (Marine Environment Monitoring Services - Marine Data Store, European Union) online database from 2009 to 2012. Variables were used on a daily temporal resolution for the circulation analysis of the basin. Colorful arrows reveal the movement direction of each whale shark on the selected date.

**Fig. 4:**
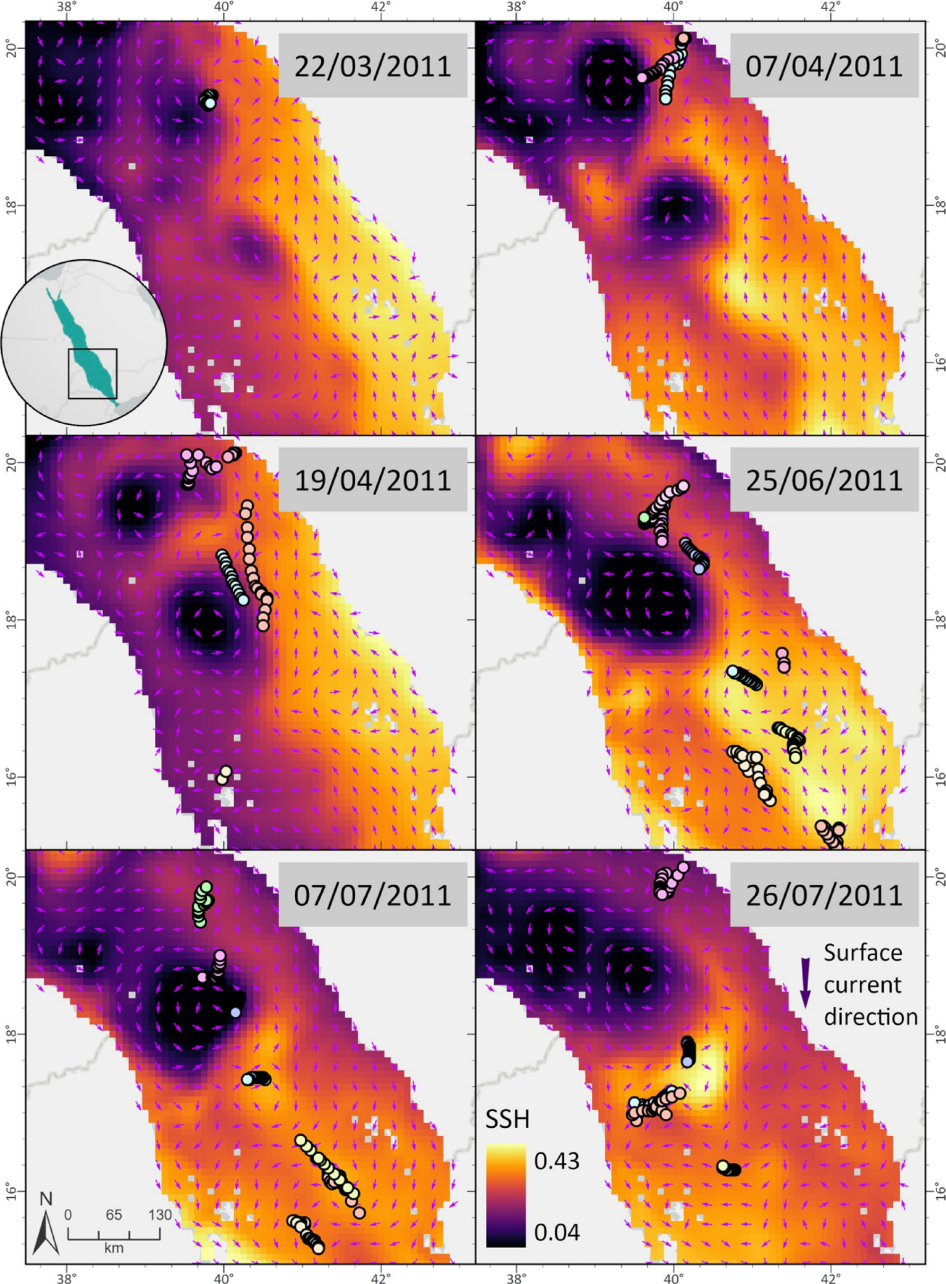
High resolution (SPOT and MK10-FL) movement track data for individual whale sharks from March 22nd until July 26th, 2011, grouped with sea surface height (SSH) circulation analysis for the Red Sea area. Individuals showcased interactions with both cyclonic (dark purple) and anticyclonic (bright yellow) eddy features along the basin, staying within its limits for several days. SSH data for the Red Sea was gathered from Copernicus (Marine Environment Monitoring Services - Marine Data Store, European Union) online database from 2009 to 2012. Variables were used on a daily temporal resolution for the circulation analysis of the basin. Colorful arrows reveal the movement direction of each whale shark on the selected date.

Tracking data revealed a potential close spatial association between whale shark horizontal movements and the presence of both anticyclonic and cyclonic mesoscale eddies in the study area. As individuals shifted between regions, their trajectories frequently aligned with the position and propagation of these eddies, suggesting that the sharks may have been actively tracking such oceanographic structures. This spatial use between eddies suggests that whale sharks could exploit different types of mesoscale features (e.g, anticyclonic and cyclonic) to optimize foraging opportunities in the oligotrophic Red Sea environment (Figs. 3 and 4).

**Table 1:** Summary of Generalized Additive Mixed Model (GAMM) of the environmental variables found to influence whale shark presence significantly (MLD = mixed layer depth; Wdir = wind direction; SST = sea surface temperature; VCUR = north-south current velocity; ). Included are the respective variable names and types, effective degrees of freedom (edf), reference degrees of freedom (Ref.df), chi-squared (Chi.sq) and p-values (p).

Variable	Type	edf	Ref.df	Chi.sq	*p*
MLD	Fixed	3.89	3.99	94.73	**< 2e-16**
Wdir	Fixed	2.71	3.00	57.76	**< 2e-16**
SST	Fixed	2.69	3.19	44.04	**< 2e-16**
VCUR	Fixed	3.54	3.89	53.07	**< 2e-16**
id	Random	20.34	25	210.84	**< 2e-16**

## Discussion

Our study suggests that juvenile whale sharks monitored between 2009 and 2012 primarily selected dynamic ocean regions along the Red Sea basin, characterized by intense currents, deep mixing, and the presence of eddies. While mixed-layer depth and north-south current velocity have not been identified as predictors of whale shark presence in previous studies, they emerged here as key drivers of movement patterns in the Red Sea population.

The movement persistence analyses have shown that individuals spend more time in the central and southern regions of the basin (characterized by lower γ_t_ values), along with some sporadic seasonal migration across the basin in the northern direction. This pattern is expected, as the Red Sea exhibits a distinct latitudinal gradient in biological productivity, with higher chlorophyll-a concentrations and primary production in the southern basin compared to the north, driven by nutrient inputs, less stratification, and circulation dynamics associated with the Gulf of Aden^25,38–40^. Remote-sensing and in-situ studies report mean chlorophyll levels of ~ 1–3 mg m^−3^ in the south versus ~ 0.2–0.45 mg m^−3^ in the north, and compiled productivity estimates further confirm higher carbon uptake rates toward the southern Red Sea^38,41^. The occupation of the northern part of the basin by this species could also be explained by the presence of permanent and semi-permanent eddies in this area^42^, formed by regional oceanographic circulation and wind patterns. As shown by Berumen et al.^18^, whale shark migration patterns in the Red Sea reveal habitat preferences based on the time of year. One possible explanation is the seasonal circulation patterns within the area, which favor mesoscale features and productivity blooms^43^. During winter (November-April), intrusive waters from the Gulf of Aden are dispersed throughout the basin, which increases productivity, primarily in the central and southern areas, by introducing high-nutrient waters from the Indian Ocean^28,29^. Subsequently, in the summer (May–October), when mesoscale eddies are more frequent, high-nutrient waters are more widespread, and upwelling occurs in multiple regions^39,44–46^. Whale shark seasonal migration based on oceanographic conditions and productive areas is common and well-known worldwide^47^.

The relatively deep (40 m and > 120 m) mixed layer, found to be the most influential variable upon the whale shark presence, relates directly to previously observed associations of this species with greater depths^48^. This layer is extremely dynamic, and its depth in the water column varies seasonally, mixing water from both shallow and deep layers, resulting in high concentrations of nutrients and organic matter^49–51^. Whale sharks require large concentrations of food uptake in order to sustain their high metabolic rates, particularly during early life stages^52–54^. Considering the oligotrophic and warm nature of the Red Sea, this basin may pose metabolic challenges for the local juvenile population, which requires constant energy acquisition. As suggested by our model, remaining within the mixed layer range appears to be more suitable for these young individuals and likely provides greater opportunities for feeding, as past studies have also identified^55^. The deepening of the mixed layer, a common phenomenon driven by various oceanographic processes^25,29,39^, may benefit young whale sharks by expanding the productive layer and enhancing opportunities for deep-water foraging. Additionally, it is well known that the Gulf of Aden Intermediate Water (GAIW) interacts with the mixed layer within the Red Sea basin, likely creating nutrient-rich hotspots at depth^25^. Future studies could evaluate the vertical behavior of the Red Sea whale sharks in relation to mixed layer dynamics to confirm whether individuals track these changes in the deep.

Wind direction was the second most significant factor correlated with the presence of juvenile sharks in the basin. Previous research conducted in the Seychelles, Papua, and the Maldives also found a strong influence of this variable on whale shark presence, as movement patterns are indirectly influenced by wind trends and shifts^56–58^. Wind trends enhance regional productivity by generating currents and dynamic features (e.g., eddies, upwellings), which disperse nutrients and connect shallow and deep habitats^25,59^. This is of most importance in the Red Sea basin, as the lack of freshwater inputs and few connections with the broader ocean drives oligotrophy quickly, reducing nutrient and chlorophyll availability. In our model, both north and east wind directions displayed stronger effects, potentially as a result of the Indian Monsoon, which blows north-eastward winds into the basin at specific times of the year^25,60^. Additionally, this regime is known to drive local upwellings, create higher eddy activities, and facilitate the advection of nutrients from the Gulf of Aden, the Red Sea’s primary nutrient source^25,42^.

As expected, sea surface temperature was significant in affecting whale shark presence, likely due to several reasons. First, the model correlation revealed that animals were mainly present above 29 °C and absent at any temperature below that limit. Sea surface temperatures in the Red Sea are among the highest for ecosystems globally, with average surface waters ranging from ~ 25.5 °C in the northern basin to ~ 29 °C in the south, and local maxima exceeding 30 °C^61^. Analyses of multi-decadal satellite and in-situ data reveal significant warming trends that exceed the global rate, along with strong spatial gradients and interannual variability across the basin^62^. It’s important to note that most animals modeled mainly occupied the central and southern areas, where we usually find higher temperature values, likely taking advantage of productive waters advected by the Gulf of Aden.

To our knowledge, this is the first study to establish a correlation between whale shark presence and eddy locations, as revealed by sea surface height trends and animal movement. In addition, the significant effects of stronger northward/southward current speeds on whale shark presence also indicate a potential correlation with eddy locations. This relationship was previously hypothesized in the Indian Ocean^14^, although it had not been fully analyzed. Given the unique environmental conditions in the Red Sea, eddies can provide excellent foraging opportunities for whale sharks, as they inject nutrients into the euphotic zone, acting as significant drivers of plankton and nutrient transportation^31,60,63^. These features connect both shallow, oligotrophic water with the deep, high-nutrient realm through convective vertical movement^64^, generating an “oasis” of phytoplankton in the otherwise nutrient-poor pelagic ocean^65,66^. By boosting local productivity through the upwelling of nutrients, lipids, and chlorophyll between deep and shallow habitats, eddies also increase the reproduction rate and fecundity of local zooplankton communities^67^. Moreover, due to the retention and advection nature, these features act like “moving biological incubators”, as they retain planktonic populations for weeks or months within their range, allowing for the development of multiple zooplankton populations, while slowly moving through the ocean^68,69^. Remaining within the vicinity of these large moving “zooplankton generator” enhances the foraging opportunities of juvenile whale sharks across shallow and deep zones, when compared to the remaining oligotrophic regions of the Red Sea. Moreover, depending on the rotation direction, cyclonic or anticyclonic, it can either deepen or reduce the mixed-layer depth, decrease or increase sea surface temperatures, and drive distinct onshore deep-water flow and offshore shelf-water flow^70–74^. These characteristics promote a localized zone of high biological productivity trapped within the eddy structure. Nonetheless, the direct correlation or influence of these mesoscale features on the local megafauna of the Red Sea is still poorly understood.

Nonetheless, it is unknown what mechanism is primarily behind how whale sharks are able to detect eddies, as this is a recently described potential relationship for the species. Here, we hypothesize that they could take advantage of several processes: (a) follow prey signals (indirect detections), by long-range chemosensory cues (i.e., dimethyl sulfide DMS), olfaction, and visual patchiness^75,76^; (b) thermal or remote physical cues, by sensing temperature gradients, following ocean currents^14^, and learning thermal eddy signatures^77^; (c) hydrodynamic cues, using mechanosensory systems (e.g., lateral line) to detect flow disturbances, wakes, or shear associated with fronts and eddies^78^; (d) learning memory and cognitive maps, as those animals are capable of remembering where productive eddies generate and repeatedly target them throughout the years^79^. Whether whale sharks use ocean currents, rely on chemical cues, or detect eddies through thermal signatures remains to be explored in future studies.

This study provides valuable insights into the modulation of whale shark behavior in response to oceanographic conditions in the Red Sea; however, several limitations should be acknowledged. Although satellite-derived oceanographic data enable broad-scale analyses across ocean basins, they would ideally be complemented by in situ measurements to better resolve fine-scale physical and biological processes within the study region. Additionally, animal tracking was conducted using multiple tag types that differ in data resolution and analytical suitability. While this diversity allows for complementary approaches, it also constrains certain analyses, particularly horizontal movement modeling when relying on lower-resolution location estimates (e.g., light-level location estimates from MK10 tags). Future studies should carefully align research hypotheses with the spatial and temporal resolution realistically achievable by each tag type. Furthermore, only a limited number of individuals with high-resolution tracking data overlapped with eddy features, restricting the strength of inferences regarding eddy-associated behavior. Although this limits statistical power, the observed patterns remain ecologically meaningful and are consistent with findings from other marine megafauna^80,81^, warranting further investigation across populations and regions. Finally, while analyses of historical tracking data are essential for understanding baseline behaviors and long-term patterns, extending this work to more recent datasets is necessary to assess whether these relationships persist under ongoing and rapid environmental change.

**Fig. 5:**
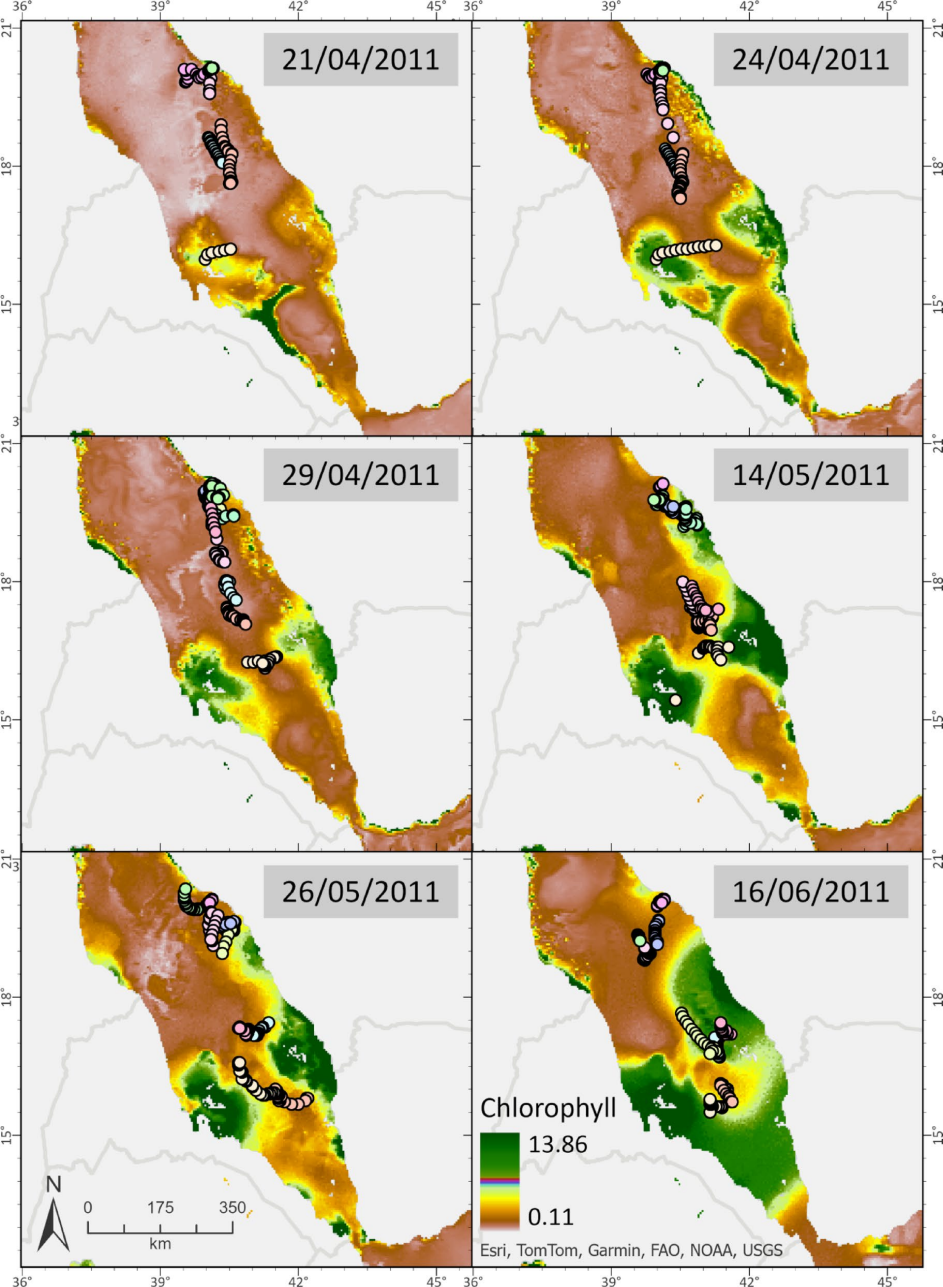
Satellite tracking of young whale shark individuals reveals a lack of direct overlap with high productivity areas within the Red Sea basin (west and east coasts), characterized by remote sensing chlorophyll content. Chlorophyll-a data for the area were gathered from Copernicus (Marine Environment Monitoring Services - Marine Data Store, European Union) online database for 2011. Variables were used on a daily temporal resolution.

Our large-scale analysis has provided a broader understanding of whale shark preferred habitats according to season and the prevailing ocean dynamics, offering additional insights into the findings of previous studies^18,20^. As past research has demonstrated, whale sharks are believed to adjust their behavior in response to local productivity, temperature variations, provisioning sites, and ocean currents^6,56,59,82–86^. Notably, while chlorophyll-a concentrations are primarily aggregated along the Red Sea coasts^87^(Fig. 5), whale sharks exhibited a preference for the basin’s axis and eddy zones, indicating a distinct behavior when compared to populations in other regions of the world. Our data further suggest that whale sharks may associate with eddies further in other regions globally, particularly under climate change scenarios. This study is the first to establish such a connection for the species within the Red Sea—a hot, hyper-saline basin often regarded as a “climate change analog” for future ocean conditions^88^. The observed pattern could inform predictions of whale shark distributions in other key habitats, such as Australia and the Galápagos.

Climate change is already reshaping marine ecosystems, driving poleward distribution shifts in mobile species^89,90^, and increasing mortality among less resilient organisms. For whale sharks, whose range is closely tied to foraging opportunities, these changes are critical. Emerging evidence, including sightings in new regions such as the southwestern Atlantic (Mesquita et al., unpubl. data), underscores their behavioral plasticity in tracking prey. We hypothesize that populations worldwide may increasingly rely on eddies as foraging hotspots, mirroring observations in the Red Sea. To support conservation efforts, future studies should integrate climate-driven ocean modeling of eddy dynamics to predict habitat suitability for this threatened species. Such approaches could prove vital in mitigating climate-induced range contractions or expansions.

**Fig. 6:**
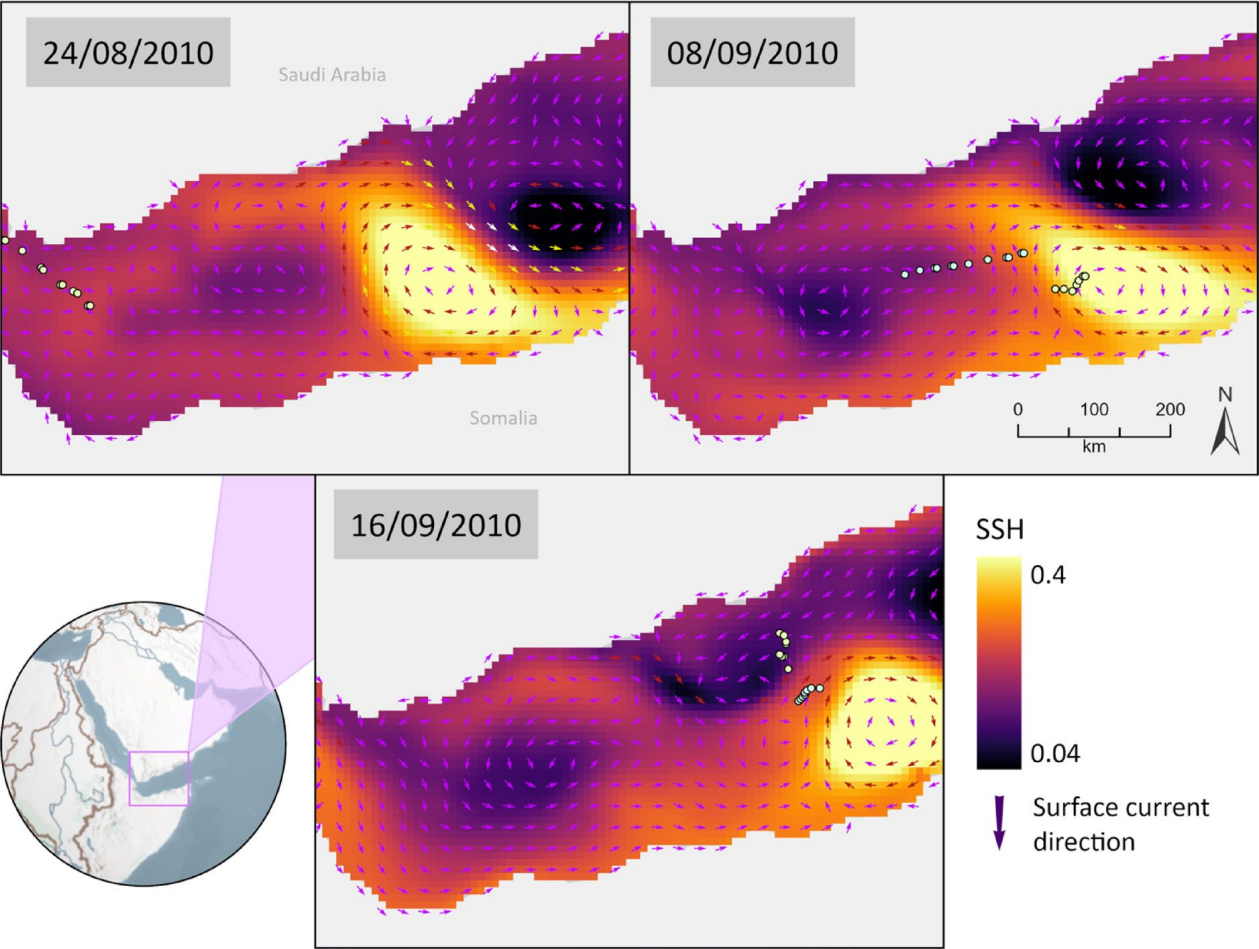
Movement tracks of two whale sharks (August–September 2010) show their migration from the Red Sea into the Gulf of Aden. Both individuals followed a persistent anticyclonic eddy (indicated by light yellow shading and clockwise-directed arrows), a recurrent oceanographic feature in this region. Sea surface height (SSH) data were obtained from the Copernicus Marine Environment Monitoring Service (CMEMS; Marine Data Store, European Union) at daily temporal resolution for circulation analysis.

**Fig. 7:**
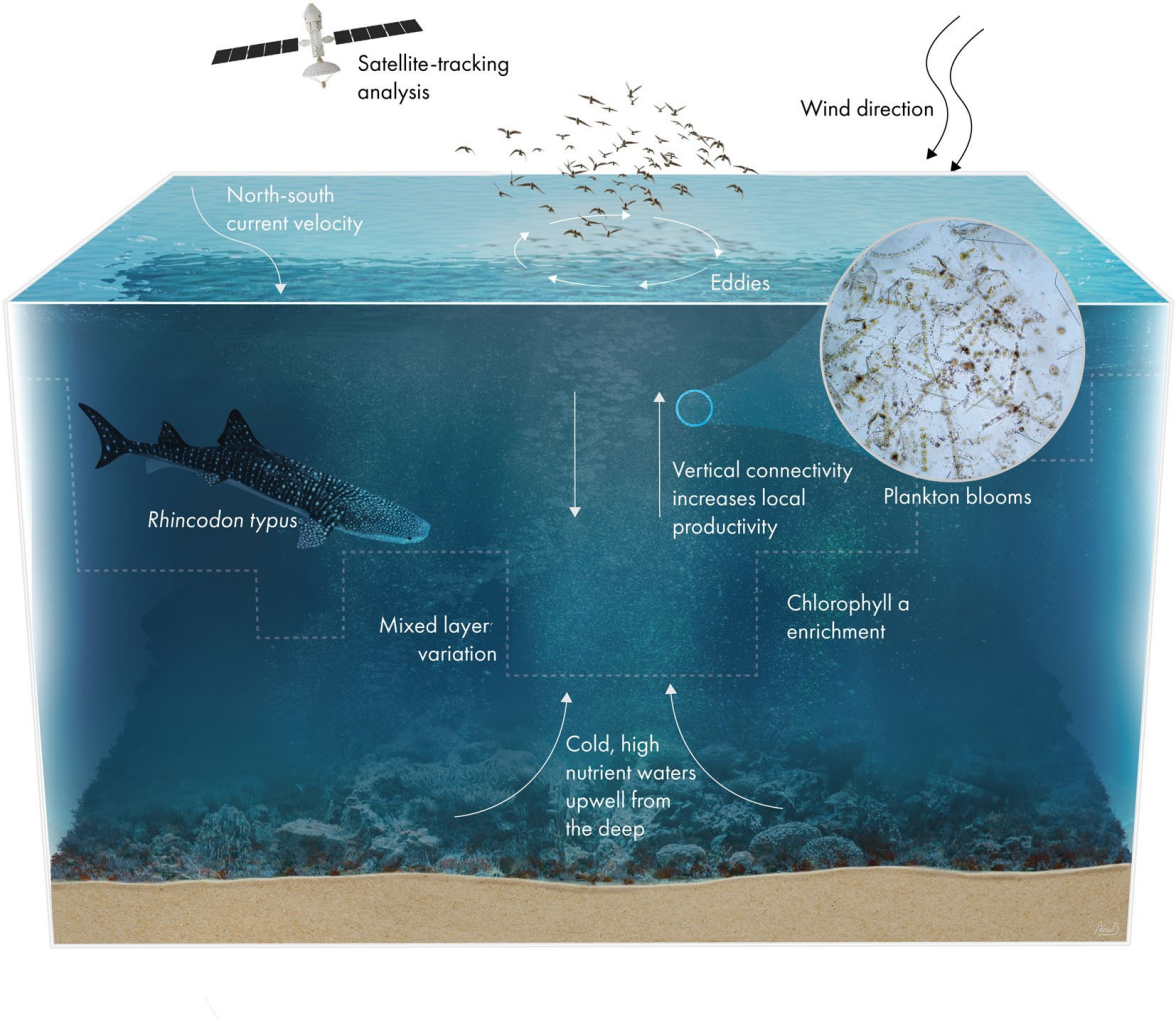
Schematic representation of key oceanographic drivers associated with whale shark (*Rhincodon typus*) occurrence in the Red Sea area. Deep mixed layers and wind- and current-generated eddy structures induce vertical connectivity between shallow and deep habitats, thereby enhancing local productivity by plankton blooms. Illustration artwork: Ana Bigio / Research Communication – KAUST.

Another application of our findings highlights the potential utility of eddies as predictors of whale shark occurrence in coastal zones adjacent to human activities, such as ports or marinas. For instance, in our study, 2 individuals migrated southward from the Red Sea into the Gulf of Aden, aggregating along the Yemeni/Somalian coast within a persistent eddy during mid-summer—a period of heightened coastal productivity (Fig. 6). Such insights are critical for national coastal management, enabling proactive measures such as seasonal closures or vessel traffic restrictions to mitigate boat strikes, a leading cause of injury and potential mortality for whale sharks^91,92^. While fatal encounters remain underreported, the scars observed on individuals underscore the severity of this threat. This knowledge can directly inform management strategies, including the designation of seasonal protected areas or adjustments to marine traffic routes. By leveraging the eddy-driven predictability of whale shark aggregations (Fig. 7), policymakers can implement targeted conservation measures to reduce human-wildlife conflicts. Seasonal eddy monitoring, coupled with dynamic ocean management, offers a scalable framework to safeguard this vulnerable species while balancing coastal economic activities.

## Conclusion

In oligotrophic basins, where nutrient availability and primary productivity are largely driven by ocean circulation and physical forcing, the high metabolic demands of juvenile whale sharks may promote behavioral adaptations that favor the use of transiently productive areas. In the Red Sea, the occurrence of whale sharks is closely associated with regions characterized by deep mixed layers, monsoon-driven winds, and northward currents that facilitate the upward transport of nutrient-rich waters, thereby supporting the energetic requirements of growth and survival. As the Red Sea is characterized by high temperatures and is often considered “the global warming ocean”, similar behavioral patterns may emerge in other whale shark populations as climate change intensifies, as they could begin relying on large oceanic features in order to survive. These features may serve as important predictors for future research on megafauna aggregation and movement. Notably, this study provides the first evidence of a potential correlation between whale shark space use and mesoscale eddies, which can enhance local productivity and increase foraging opportunities. These findings highlight the importance of dynamic oceanographic features in shaping whale shark habitat use and underscore their potential role in sustaining populations under future environmental change. While our findings highlight a significant relationship with three-dimensional ocean features, it is essential to consider the regional dynamics and the influence of large-scale climate events. Additionally, as this study relies on modeled and historical data, future research should validate these patterns through in situ observations using instruments like gliders and CTDs to measure local features directly.

## Supplementary Information

Below is the link to the electronic supplementary material.

Supplementary Material 2

Supplementary Material 1

Supplementary Material 3

## Data Availability

All data generated or analyzed in this study are available from the corresponding author upon reasonable request.

## Abbreviations

IUCN: International Union for Conservation of Nature
CITES: Convention on International Trade in Endangered Species
SPOT: Satellite Positioning and Temperature Tag
GAMM: Generalized Additive Mixed Model
BRAN: Bluelink Reanalysis Model
SST: Sea Surface Temperature
SSH: Sea Surface Height
MLD: Mixed Layer Depth
Wspd: Wind Speed
Wdir: Wind Direction
VCS: Vertical current speed
HCS: Horizontal current speed
VCUR: North South Current Velocity
UCUR: West East current
WCUR: Bottom surface current
ChlA: Chlorophyll-a
AIC: Akaike Information Criterion
ANOVA: Analysis of Variance
GAIW: Gulf of Aden Intermediate Waters
