# Supplementary figures and images for: Flow-driven patterns of whale shark movement in the Red Sea
Source: Sci Rep. 2026 Apr 2;16:15773. doi: 10.1038/s41598-026-45029-8 (PMC13194706; doi:10.1038/s41598-026-45029-8)

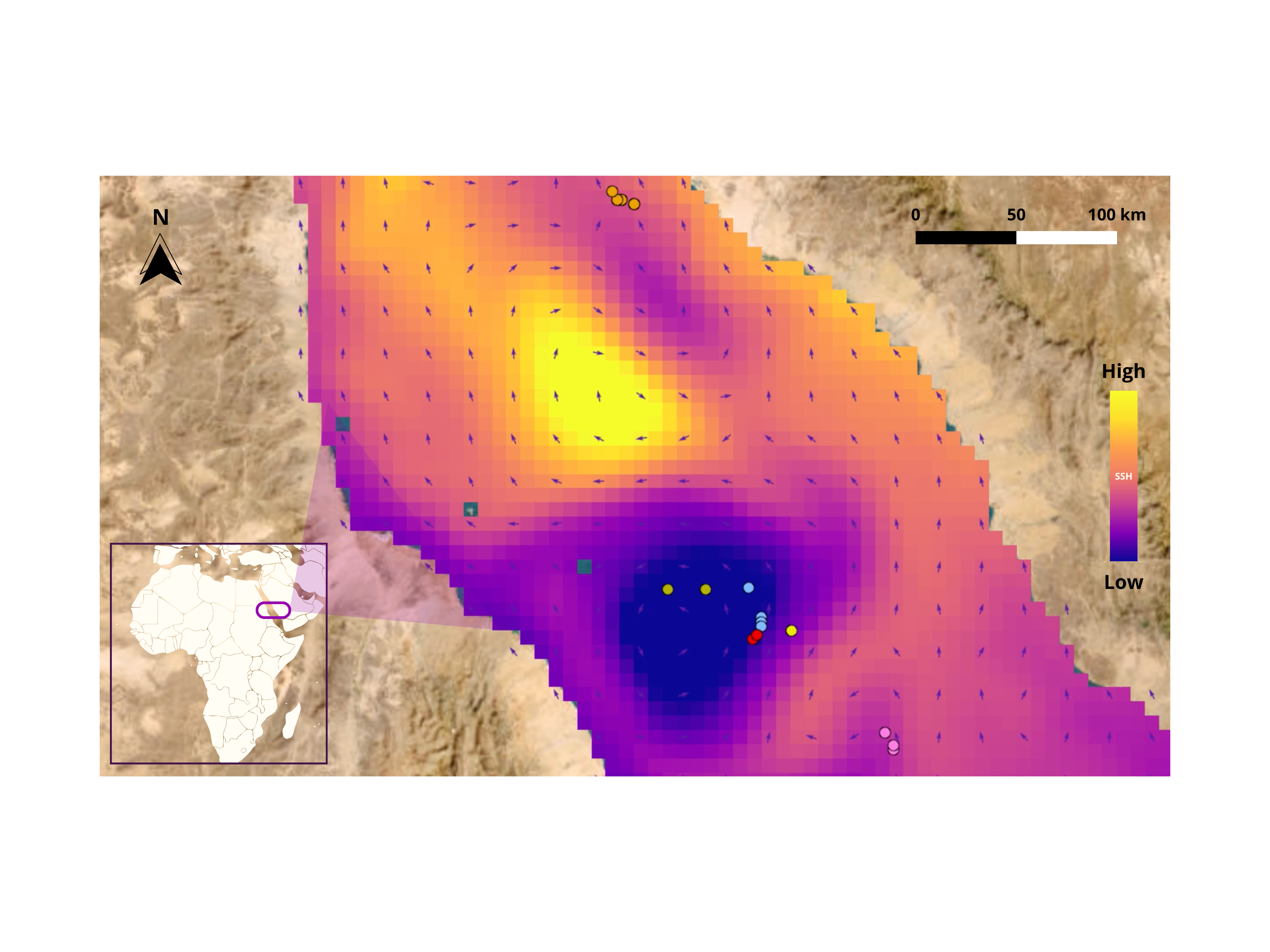

Supplement: Supplementary file 1 — Supplementary Material 2 [file 41598_2026_45029_MOESM1_ESM.zip › Video_1_Still.png]

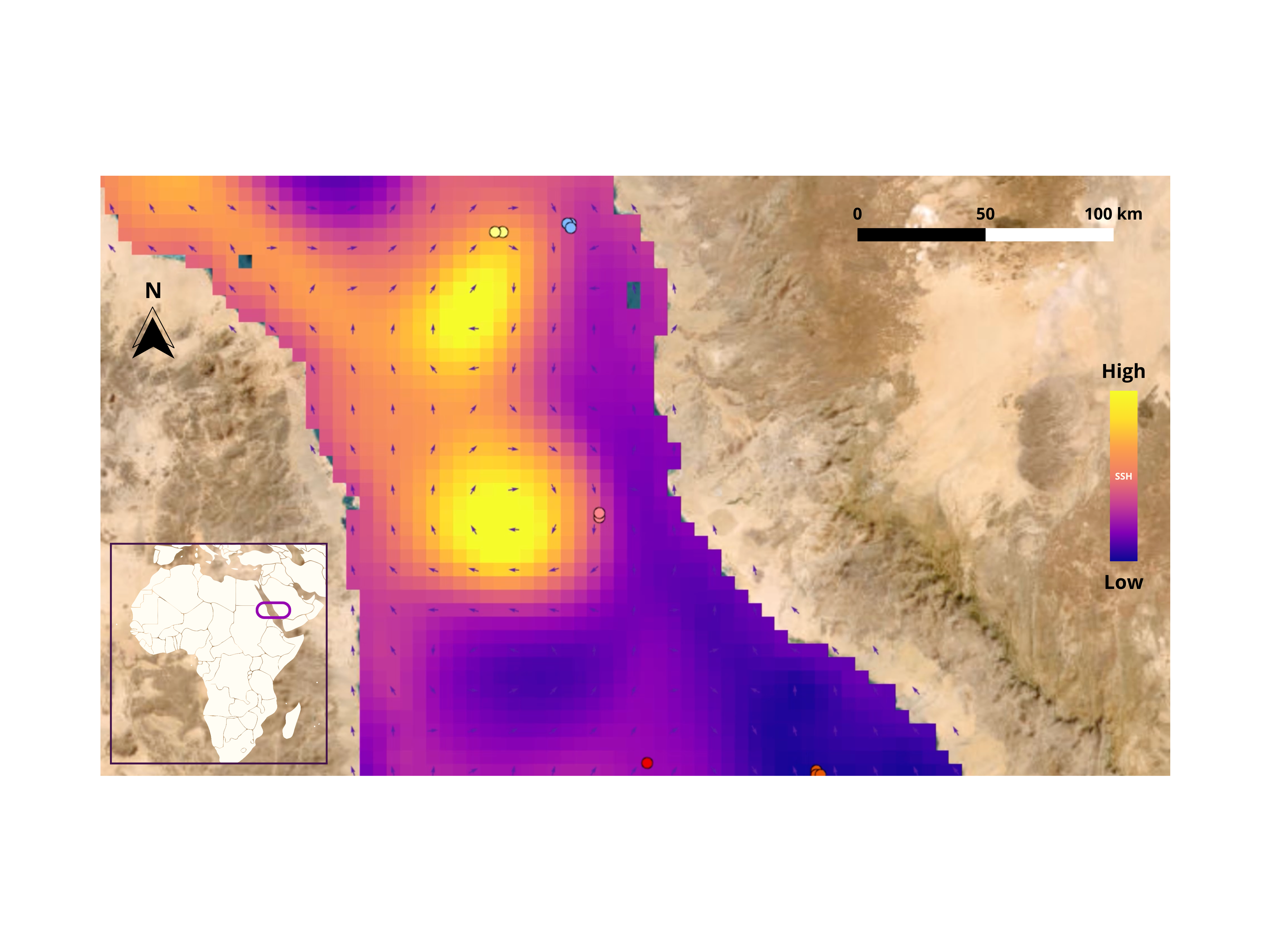

Supplement: Supplementary file 1 — Supplementary Material 2 [file 41598_2026_45029_MOESM1_ESM.zip › Video_2_Still.png]

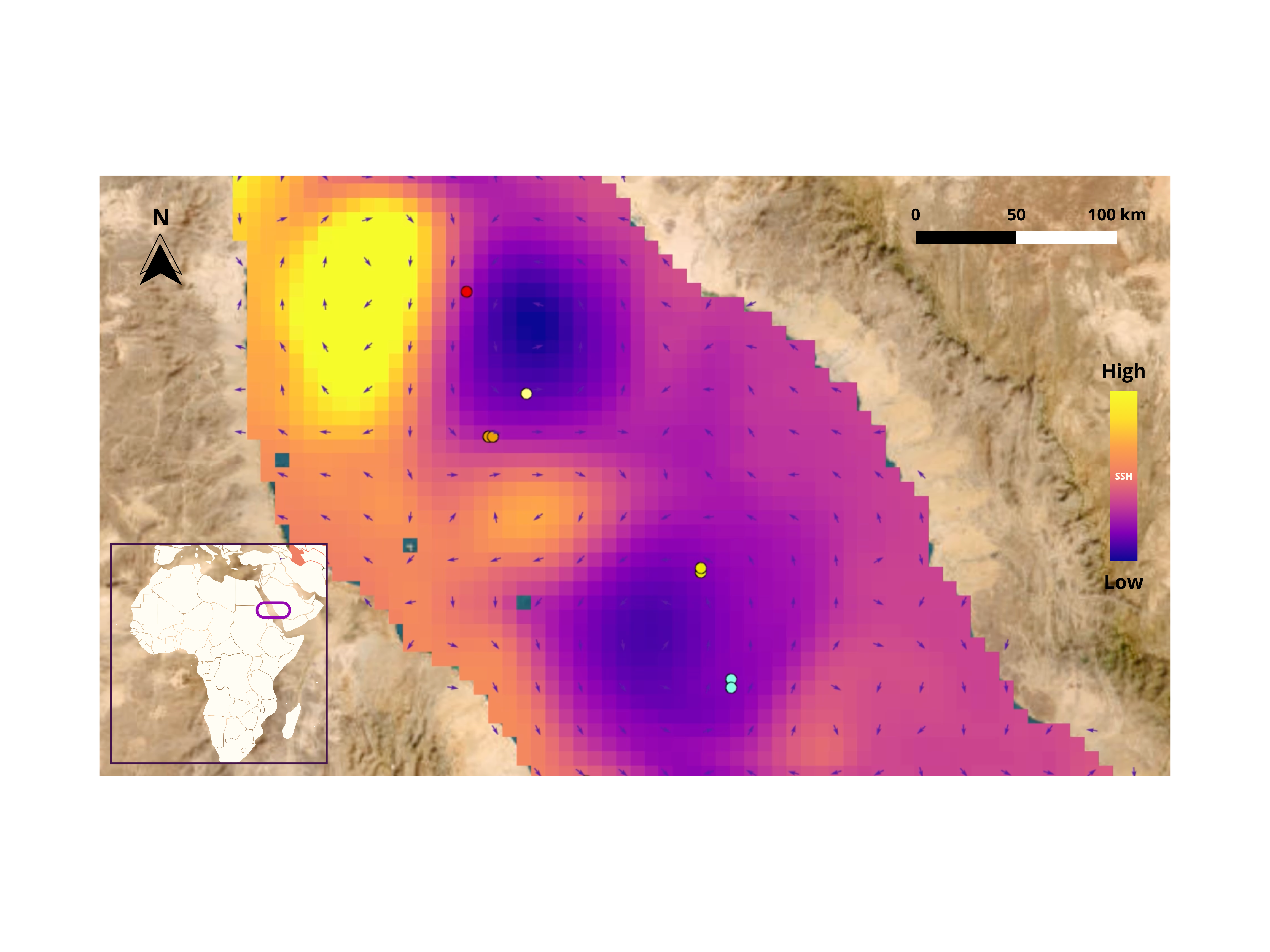

Supplement: Supplementary file 1 — Supplementary Material 2 [file 41598_2026_45029_MOESM1_ESM.zip › Video_3_Still.png]
