# Supplementary material for: Flow-driven patterns of whale shark movement in the Red Sea
Source: Sci Rep. 2026 Apr 2;16:15773. doi: 10.1038/s41598-026-45029-8 (PMC13194706; doi:10.1038/s41598-026-45029-8)
Supplement: Supplementary file 2 — Supplementary Material 1 [file 41598_2026_45029_MOESM2_ESM.pdf]

Supplemental Material

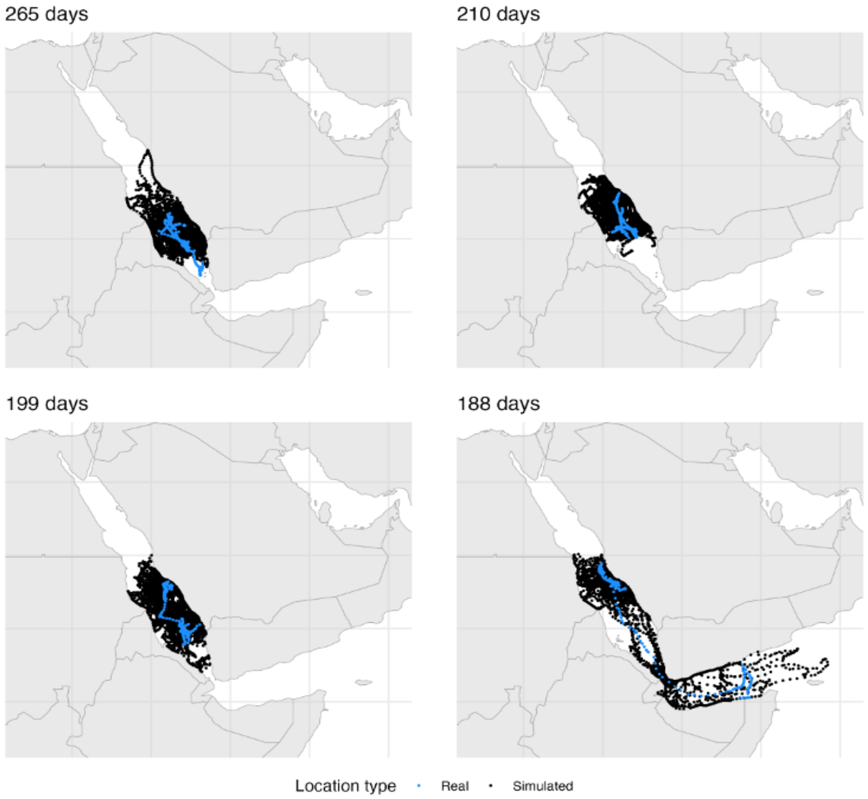

Figure S1. Examples of real (blue points) and simulated tracks (black points) for four individual whale sharks tracked between 188 and 265 days in the Red Sea area.

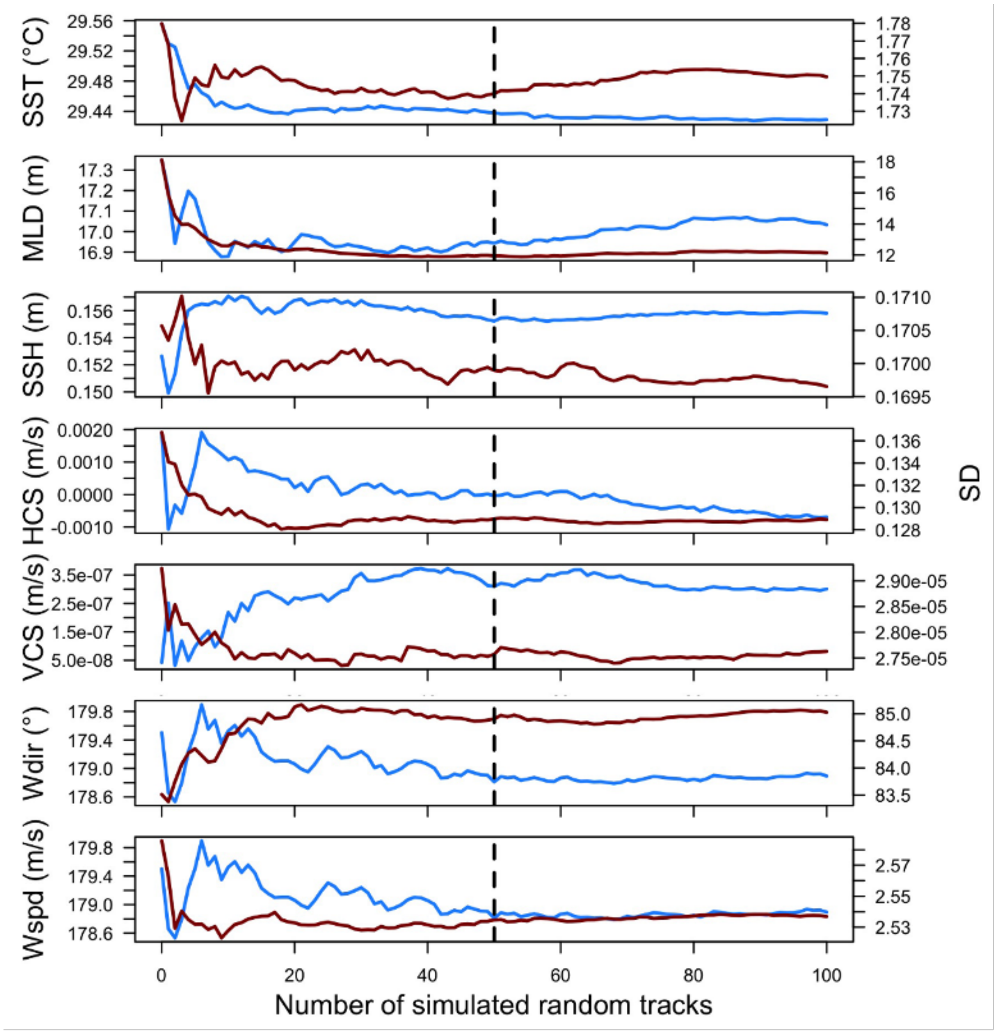

Figure S2. Mean (blue line) and standard deviation (SD; red line) variations for all environmental variables (SST = sea surface temperature; MLD = mixed layer depth; SSH = sea surface height; HCS = horizontal current speed; VCS = vertical current speed; Wdir = wind direction; Wspd = wind speed) as a function of cumulative number of simulated random tracks used.

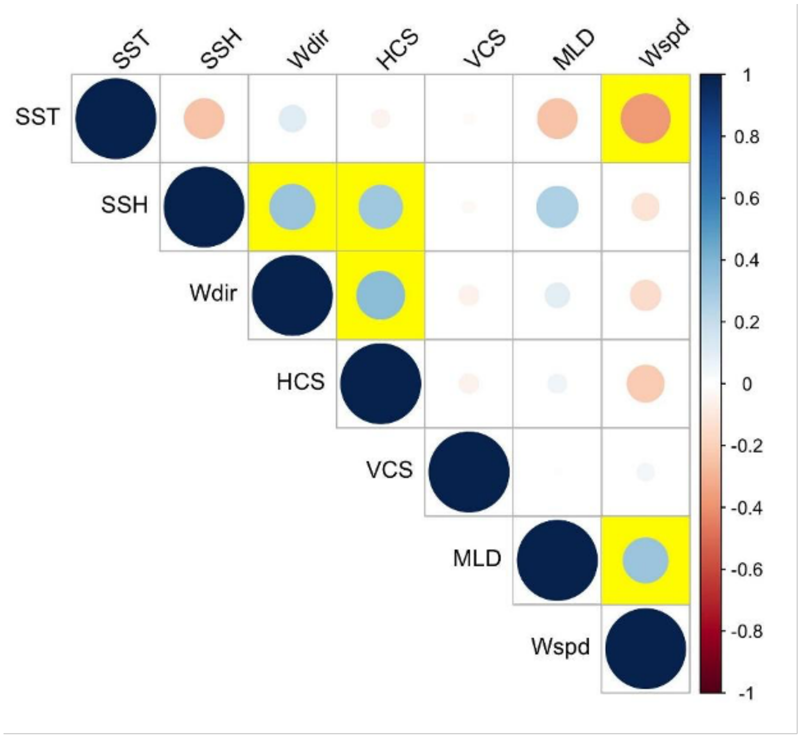

Figure S3. Correlation matrix between the candidate environmental variables (SST = sea surface temperature; MLD = mixed layer depth; SSH = sea surface height; HCS = horizontal current speed; VCS = vertical current speed; Wdir = wind direction; Wspd = wind speed) included in the modeling approach. Significantly correlated variables are highlighted in yellow.

Table S1. Variable selection procedure for the Generalized Additive Mixed Model of environmental parameters influencing whale shark presence. The final model is highlighted in bold.

Model	AIC	ΔAIC	wAIC
NULL	7202.16	181.65	<0.0001
MLD	7083.01	62.50	<0.0001
SSH*			
SST	7193.38	172.87	<0.0001
Wdir	7176.45	155.94	<0.0001
Wspd*			
VCS	7157.86	137.35	<0.0001
HCS	7186.77	166.26	<0.0001
MLD + SST	7065.25	44.74	<0.0001
MLD + Wdir	7056.39	35.88	<0.0001

MLD + VCS	7061.78	41.27	<0.0001
MLD + HCS	7073.83	53.32	<0.0001
MLD + Wdir + SST	7042.24	21.73	<0.0001
MLD + Wdir + VCS	7040.54	20.03	<0.0001
**MLD + Wdir + VCS + SST**	**7020.51**	**0**	**0.9999**

(*) Variable discarded due to lack of statistical significance
