# Supplementary material for: Flow-driven patterns of whale shark movement in the Red Sea
Source: Sci Rep. 2026 Apr 2;16:15773. doi: 10.1038/s41598-026-45029-8 (PMC13194706; doi:10.1038/s41598-026-45029-8)
Supplement: Supplementary file 3 — Supplementary Material 3 [file 41598_2026_45029_MOESM3_ESM.docx]

Text for videos

Video_1 text: Juvenile whale sharks (*Rhincodon typus*) follow both an anticyclonic and a cyclonic eddy located in the central Red Sea basin from the 26^th^ of July to the 11^th^ of August, 2010. Individuals preferred the anticyclonic features’ peripheries and the cyclonic features’ cores.

Video_2 text: Juvenile whale sharks (*Rhincodon typus*) follow two anticyclonic eddies located in the central Red Sea basin in May 2010 after leaving an aggregation area (Al Lith, central Red Sea). Individuals revealed preferences for the anticyclonic features’ peripheries, where productivity is elevated compared to the core.

Video_3 text: Juvenile whale sharks (*Rhincodon typus*) follow two cyclonic eddies located in the central/southern Red Sea basin from the 13^th^ of November until the 18^th^ of December, 2010. Individuals revealed preferences for the cyclonic features’ cores, where productivity is elevated compared to the peripheries.
